# Dimensionless, Scale Invariant, Edge Weight Metric for the Study of Complex Structural Networks

**DOI:** 10.1371/journal.pone.0131493

**Published:** 2015-07-14

**Authors:** Luis M. Colon-Perez, Caitlin Spindler, Shelby Goicochea, William Triplett, Mansi Parekh, Eric Montie, Paul R. Carney, Catherine Price, Thomas H. Mareci

**Affiliations:** 1 Department of Physics University of Florida, Gainesville, Florida, United States of America; 2 Department of Biology University of Florida, Gainesville, Florida, United States of America; 3 Department of Chemistry University of Florida, Gainesville, Florida, United States of America; 4 Department of Biochemistry and Molecular Biology University of Florida, Gainesville, Florida, United States of America; 5 Department of Pediatrics University of Florida, Gainesville, Florida, United States of America; 6 Department of Natural Science, University of South Carolina Beaufort, Bluffton, South Carolina, United States of America; 7 Department of Clinical Heath Psychology University of Florida, Gainesville, Florida, United States of America; Shenzhen Institutes of Advanced Technology, CHINA

## Abstract

High spatial and angular resolution diffusion weighted imaging (DWI) with network analysis provides a unique framework for the study of brain structure *in vivo*. DWI-derived brain connectivity patterns are best characterized with graph theory using an edge weight to quantify the strength of white matter connections between gray matter nodes. Here a dimensionless, scale-invariant edge weight is introduced to measure node connectivity. This edge weight metric provides reasonable and consistent values over any size scale (e.g. rodents to humans) used to quantify the strength of connection. Firstly, simulations were used to assess the effects of tractography seed point density and random errors in the estimated fiber orientations; with sufficient signal-to-noise ratio (SNR), edge weight estimates improve as the seed density increases. Secondly to evaluate the application of the edge weight in the human brain, ten repeated measures of DWI in the same healthy human subject were analyzed. Mean edge weight values within the cingulum and corpus callosum were consistent and showed low variability. Thirdly, using excised rat brains to study the effects of spatial resolution, the weight of edges connecting major structures in the temporal lobe were used to characterize connectivity in this local network. The results indicate that with adequate resolution and SNR, connections between network nodes are characterized well by this edge weight metric. Therefore this new dimensionless, scale-invariant edge weight metric provides a robust measure of network connectivity that can be applied in any size regime.

## Introduction

A more complete understanding of brain function requires detailed information about which brain regions are structurally connected and how the strength of these connections relates to function [1, 2]. Recently developed innovative methods of determining brain structure *in vivo* [3–9] have shown that diffusion-weighted magnetic resonance imaging (DWI) can provide enhanced gray matter (GM) and white matter (WM) contrast, due to local tissue anisotropy [10], and also allows streamline modeling of WM fiber structure in each voxel [11], particularly in regions where fibers “kiss” or cross. Using DWI tractography techniques [12] and network analysis [13], graph theory models of brain WM connectivity networks [14] *in vivo* can be created in which GM anatomical regions correspond to network nodes and WM fibers correspond to network edges connecting nodes.

Recent interest in brain connectivity has focused largely on the large-scale cortical structure of the brain [15–22]. In addition to cortical analysis, graph theory has been applied to understand the relation between structural connections and brain development [23], as well as pathological states like epilepsy [24], schizophrenia [25], Alzheimer’s [26], Parkinson’s disease [27], and multiple sclerosis [28].

**Fig 1:**
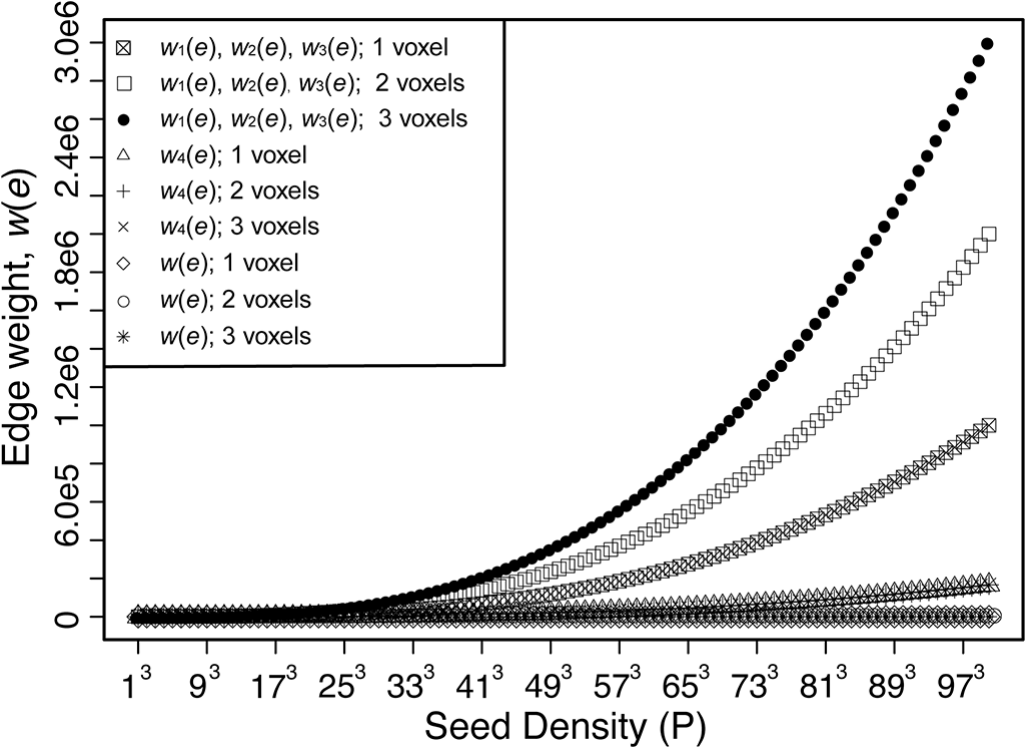
Edge weight, *w*(*e*), values as seed density increases for a 3D straight edge. For this calculation, the image resolution, d, is taken to be 1, which results in a voxel volume of 1, and a surface area of 6. The calculation of *w*_2_(*e*) assuming an FA value of 1. These assumptions made the value for *w*_1_(*e*), *w*_2_(*e*), and *w*_3_(*e*) of Table 1 equal. For a different value of FA, *w*_2_(*e*) behaves similarly to *w*_1_(*e*) scaled by the appropriate FA value along the track. As described by Hagmann et. al. [16], *w*_4_(*e*) removes the length dependence. However, *w*_4_(*e*) still displays a divergent asymptote as seed density increases. The edge weight proposed in this paper, *w*(*e*) (Eq 1), displays a constant value of 1/6 for all seed densities.

Most MRI studies of brain networks use binary graph metrics of connectivity, but recently edge weights have been introduced as a way of quantifying the strength of connections [29, 30]. An ideal edge weight metric should quantify the strength of connection in a logical manner that can be applied to a brain of any size (e.g. rodents to humans) and be independent of image resolution. Also the edge weight should be dimensionless in order to represent an analytical weighting factor that is independent of the measured quantifies which fits into an expression of connection strength between nodes [31]. Currently available DWI-based edge weight metrics are summarized in Table 1. Only edge weight 1, 2, and 5 (the latter introduced in this manuscript) are dimensionless, while edge weights 3 and 4 have dimensions that are the inverse third power of distance, so they depend on brain size. In addition, edge weights 1–4 all depend on image resolution. Edge weight 2 depends on the value of fractional anisotropy (FA); however, FA may not be an adequate measure of connectivity strength because it does not adequately parameterize the underlying tissue structure in regions of complex fiber structure and is affected by acquisition parameters (e.g. b-values, number of gradient directions). In addition, the values of edge weights 1–4 diverge as track seeding point densities increase (see Fig 1 and discussion in the Methods). However high seed densities (e.g. > 40 seed points per voxel [29]) are needed to increase reliability of network metrics. So a scheme for calculating edge weights is needed that provides a reliable measure of connectivity strength, which converges to a stable value as the seed density increase, and is independent of length scale, image resolution, and the tractography scheme used to estimate WM fibers.

**Table 1:** Edge weights, *w*(*e*), derived from DWI tractography. The edge weight, *w*_1_(*e*_*ij*_), introduced by Li, et.al., is defined by *S*_*ij*_, which is the number of set of streamlines connecting node *i* to *j*. The edge weight, *w*_2_(*e*_*ij*_) (introduced by Lo, et.al.), depends on *S*_*ij*_ and *FA*, which is the average fractional anisotropy value from all voxels making up the edge. The edge weight, *w*_3_(*e*_*ij*_) (introduced by Buchanan et.al.), depends on *S*_*ij*_ and the volume of the connected nodes, *V_x_*, when *x* = *i* or *j*. The edge weight, *w*_4_(*e*)) (introduced by Hagmann et.al.) depends on the inverse of *l*(*f*), which is the length of the streamline *f* (in units of distance) from the set of all streamlines connecting node *i* to *j*, and *A_i_* and *A_i_* are the surface areas of node *i* and *j*. Finally the edge weight described in this manuscript (*w*(*e_ij_*), 5th row) is defined over the set *M* of voxels making up the edge, *m* is a voxel from set *M*, *P_voxel_* is the number of seed points per voxel, *p* is a seed point out of all *P_voxel_* from voxel *m*, *V_voxel_* is the image voxel volume, *A_i_* is the surface area of node i and *f_m,p_* corresponds to a streamline originating from voxel *m* and seed point *p*. Generally, edge weights use in literature are some variation of weighting with the number of streamlines, FA or 1/ *l*(*f*).

Edge weight		Characteristics
1	*w*_1_(*e*_*ij*_) = |*S*_*ij*_|,[32]	Dimensionless, but biased towards longer lengths and higher resolution yields larger edge weights for a tract of a fixed volume.
2	*w*_2_(*e*_*ij*_) = FA*|*S*_*ij*_|,[26]	Dimensionless, but biased towards longer lengths and FA does not characterize adequately complex fiber structures. Higher resolution yields larger edge weights for a tract of a fixed volume.
3	$w_{3}(e_{ij})=\frac{2}{V_{i}+V_{j}}\left|S_{ij}\right|,$ [30]	Dimensions of [length]^-3^, increases as the cube of seed density and is biased towards longer edges. Higher resolution yields larger edge weights for a tract of a fixed volume.
4	$w_{4}(e_{ij})=\frac{2}{A_{i}+A_{j}}\sum_{f\in S_{ij}}\frac{1}{l(f)},$ [16].	Dimension of [length]^-3^, removes tract length dependence, but increases as the cube of seed density. Higher resolution yields larger edge weights for a tract of a fixed volume.
5	$w(e_{ij})=\left(\frac{V_{voxel}}{P_{voxel}}\right)\left(\frac{2}{A_{i}+A_{j}}\right)\sum_{m=1}^{M}\sum_{p=1}^{P_{voxel}}\frac{\chi_{R}(f_{m,p})}{l(f_{m,p})}$	Dimensionless, does not depend on tract length or image resolution, and converges to a fix value as seed density increases (introduced in this manuscript).

In this study, a dimensionless, scale-independent edge weight measure of node connectivity is presented, which is derived from diffusion-weighted tractography. This new edge weight is based on the previously defined edge weight 4 in Table 1 [15] and provides an edge weight that is dimensionless, independent of brain size and resolution, and relatively independent of the parameters used for diffusion acquisition and tractography (e.g. seed density). As described in the Methods section, this new edge weight introduces the use of an indicator function to restrict the streamlines of the edge to only those originating from the WM voxels in the edge between connected GM nodes. To illustrate the application of this edge weight to network analysis, this new metric is used to quantify connectivity between the major structures in sub-cortical network of the limbic system in the mesial temporal lobe (TL). Abnormal connectivity in the TL is thought to be related to disorders, such as epilepsy [33], and changes in the connectivity between the following major structures of the TL are theorized to be the source of epilepsy emergence [34]: the hippocampus (HC), amygdala (AM), thalamus (TH) and entorhinal cortex (EC). Therefore developing a method for studying the network connectivity of brain regions, like these limbic structures, using graph theory may aid in understanding the development and progression of epilepsy and other neurological disorders.

## Methods

### 2.1. Edge Weight

To represent structural connectivity in the brain as a complex network, WM fibers connecting anatomical GM structures may be described as a graph of edges connecting nodes. Throughout the rest of this manuscript, ‘fibers’ calculated with DWI tractography will be referred to as streamline and the real WM fibers in the brain will be referred to simply as fibers. Streamlines originate from track seeding points within a voxel of interest and the total number of streamline in an edge comes from two sources: the number of seeds per voxel and the number of voxels that make up the edge. For the successful creation of a network representation of the brain, an edge weight metric is needed that provides a stable, dimensionless value, that is independent of length scale, image resolution (as long as sufficient information is obtained at a given resolution to characterize the fiber), and the tractography seeding used to estimate WM fibers. However, current DWI-based edge weight metrics (see Table 1) diverge as the seeding density increases, as shown in Fig 1. For this simulation, edge weights 1 through 5 were calculated for two single isotropic-voxel nodes connected by a straight edge. For simplicity in this simulation, the voxel width (in arbitrary units) is set to d = 1, node surface area to A = 6d^2^ = 6, and the voxel isotropic volume to d^3^ = 1. As the seed density increases, the values of edge weight 1 through 4 diverge because the number of streamline calculated is directly proportional to the seed density. This behavior holds true for any edge geometry, not just the simple geometry of this illustration, so the value of these edge weights depends on the scheme for seeding fiber tracts.

To determine the connectivity strength of an edge, a dimensionless, scale-invariant edge weight [35] is proposed below in Eq 1, which is based on the previously defined edge weight 4 [15].

$$w(e_{ij})=\left(\frac{V_{voxel}}{P_{voxel}}\right)\left(\frac{2}{A_{i}+A_{j}}\right)\sum_{m=1}^{M}\sum_{p=1}^{P_{voxel}}\frac{\chi_{R}(f_{m,p})}{l(f_{m,p})},$$ (1)

where χ_*R*_ is an indicator function defined below. Relative to edge weight 1 through 4 in Table 1, this proposed edge weight removes the dependence on image resolution, and tractography seeding density, by normalizing the edge weight by the ratio of the voxel volume, *V*_*voxel*_, and the number of seeds per voxel, *P*_*voxel*_. As introduced by Hagmann, et al. [15], *A*_*n*_ is the surface area of the *n* ^*th*^ node, which provides a scaling that favors edge weights with more streamline connections for a given node surface area, while the inverse sum over streamline length makes the edge weight independent of the length of the streamlines. For the edge weight proposed in Eq 1, the first sum is over the number of voxels, *M*, in the edge, and the second sum is over the seed points in each voxel, where *l*(*f*_*m*,*p*_) is the length of the streamline originated from seed point *p* in voxel *m*. Using this formulation, the value of the edge weight will be unchanged as spatial resolution changes, since *V*_*voxel*_ and number of voxels, *M*, will change in the opposite sense by the same factor as the resolution changes. Therefore this edge weight formulation is independent of resolution.

**Fig 2:**
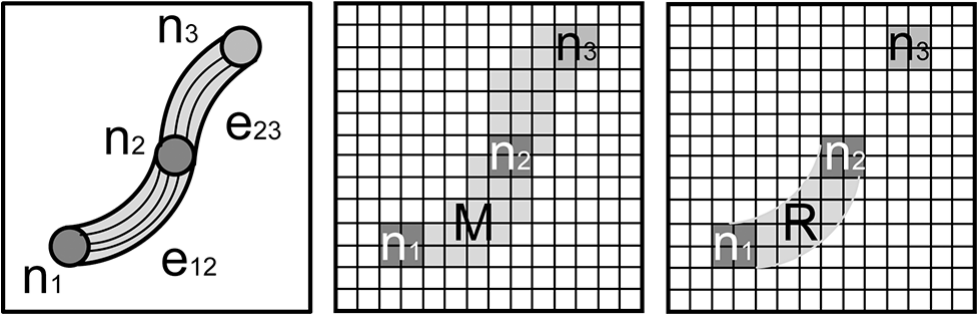
Scheme for streamline filtering. **In the left diagram, three nodes (n**_**1**_ **, n**_**2**_ **and n**_**3**_ **) are shown connected by two edges, e**_**12**_ **and e**_**23**_ **.** In the center diagram, the nodes of interest, n_1_ and n_2_, are connected by M voxels. In the right diagram, region R contains the seed points that contribute to the desired edge weight, e_12_.

To ensure an accurate estimation of WM fiber structure throughout the brain, streamlines are calculated from a seed points in every voxel within the entire brain. However, streamlines in an edge may originate from voxels outside the edge, which will result in overestimating the edge weight. The selection of only the streamlines directly connecting nodes of interest requires an appropriate WM and GM segmentation in order to only seed WM tract voxels within the edge of interest. Selecting the appropriate WM voxels requires *a priori* knowledge of the WM tract to sufficiently determinate the appropriate WM and GM boundaries; however in most cases, such segmentation is problematic. Therefore, a more general approach is taken in this work. To isolate only the streamlines forming the edge, streamline-fiber filtering is performed, with the indicator function of Eq 1, to remove streamlines originating from voxels external to the edge. To filter these streamlines from the set of all streamlines that may travel thought the set of *M* edge voxels, only the streamlines that meet the following two criteria are retained: 1) Originate from seed points in the set of *M* edge voxels and 2) directly connect the nodes (i.e., no additional nodes are found in the fiber path). Within the set of all seed points, *M x P*, in the edge, these criteria define a subset of seed points, *R*, located at positions *x*_*R*_, *y*_*R*_, and *z*_*R*_ in the *M* voxels used to calculate edge weight. Therefore, the inclusion of the indicator function,

$$\chi_{R}(f_{m,p})=\{\begin{array}{c} 1,\;f_{m,p}\;\in \;R\\ 0,\;f_{m,p}\;\notin \;R \end{array}\;,$$ (2)

will ensure that only the streamlines directly connecting these nodes are included, and eliminates spurious streamlines that do not originate from seed points in the edge. The use of this indicator function can be illustrated using the idealized structure diagrammed on the left of Fig 2, where only streamlines originating in the WM regions (light gray) and connecting the nodes (darker gray) will be included in the calculation of the edge weight and all other streamlines will be discarded. In the voxelized image of this structure, shown in middle diagram of Fig 2, the nodes (darker gray) of interest, *n*_*1*_ and *n*_*2*_, are connected by a set of *M* voxels (light gray) that define the edge, *e*_*12*_, between these two nodes. The indicator function will filter these streamlines to include only those shown in the diagram on the right in Fig 2 and eliminate the streamlines running through *e*_*12*_ but originated from the voxels in the edge between nodes 2 and 3.

**Fig 3:**
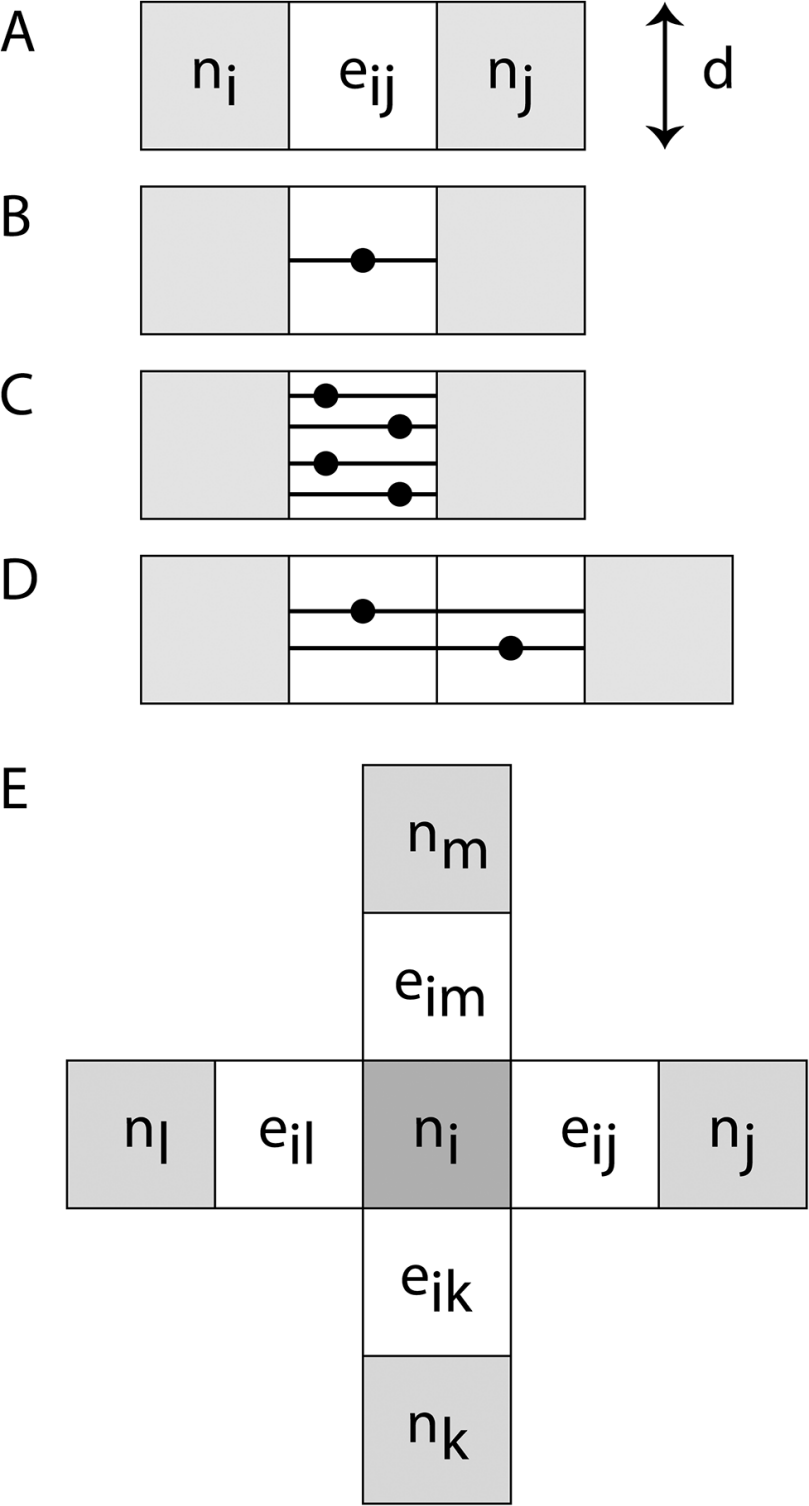
Two-dimensional WM fibers are contained within the edge, e_ij_ (white pixels), connecting nodes (gray pixels), n_i_ and n_j_, as labeled in Part A. For straight-line edges, the fiber lies within pixels of the edge. The streamline tracks are shown in pixels of the edge performed with (B) one seed-per-pixel and (C) four seeds-per-pixel in a single-pixel edge, and (D) one seed-per-pixel in a two-pixel edge. (E) A fully connected 2D network with straight edges on each face of a central node, n_i_ (dark gray), connected to four other nodes (light gray), each through a single fiber (white) similar to the fiber shown in part B-D.

The properties of this edge weight formulation, that result in independence from spatial resolution and seed density, can be most easily visualized with a simple example of two-dimensional connectivity. For application in two-dimensions, the edge weight in Eq 1 can be modified by replacing *V*_*voxel*_ with pixel area, *A*_*pixel*_, and using the node perimeter, rather than node surface area, in the calculation. The simple two-dimensional edge is illustrated in part A of Fig 3, where the connected nodes (gray boxes) are single pixels of width *d*, area *d* ^*2*^, and perimeter 4*d*. In part B, the number of seeds-per-pixel, *P*_*pixel*_, equals 1 and results in only one streamline connecting the nodes with a fiber length of *d*, which results in a dimensionless two-dimensional edge weight value of 1/4. In part C, *P*_*pixel*_ equals 4 giving four streamlines; however, the edge weight normalization factor (*A*_*pixel*_ */ P*_*pixel*_) results in edge weight value of 1/4. This result is consistent for any number of seeds-per-pixel in the edge, as shown in the examples in parts B and C. In part D, the edge consists of two pixels connecting the nodes with *P*_*pixel*_ = 1. In this case, the streamline length would be 2*d*, but since all the pixels in the streamline path are seeded, a total of two streamlines would connect the nodes. Therefore, the two-dimensional edge weight is still 1/4. For two-dimensional streamlines directly connecting identical nodes through a face (as in Fig 3) with any number of pixels in a straight edge, the edge weight value is 1/4. Therefore the edge weight result is independent of the number of pixels that make up the streamline and the number of seeds-per-pixel. In a straightforward manner, this approach can be extended to the three-dimensional case to show that the edge weight of Eq 1 is dimensionless, and independent of resolution and seed density.

The relationship between nodes within a network is central to understanding network properties. A measure of particular importance is the connectivity strength of any node in a weighted graph [31], which is defined by

$$s(n_{i})=\sum_{i\neq j}w(e_{ij}).$$ (3)

In this equation, *w*(*e*_*ij*_) is the edge weight (Eq 1) connecting node *i* to node *j*. The node strength in Eq 3 provides insight into the topological structure of a graph and allows the estimation of statistical properties within the architecture of weighted networks [36]. Returning to the two-dimensional case, a node fully connected with straight edges on each face (4 edges, each with an edge weight of 1/4), as the one shown in part E of Fig 3, the connectivity strength of central node, *n*_*i*_, in this network is equal to 1. This illustrates a useful property of the normalized weight edge defined in Eq 1, since the resulting connection strength has a logical value of 1 for a node fully connected in manner illustrated in part E.

**Fig 4:**
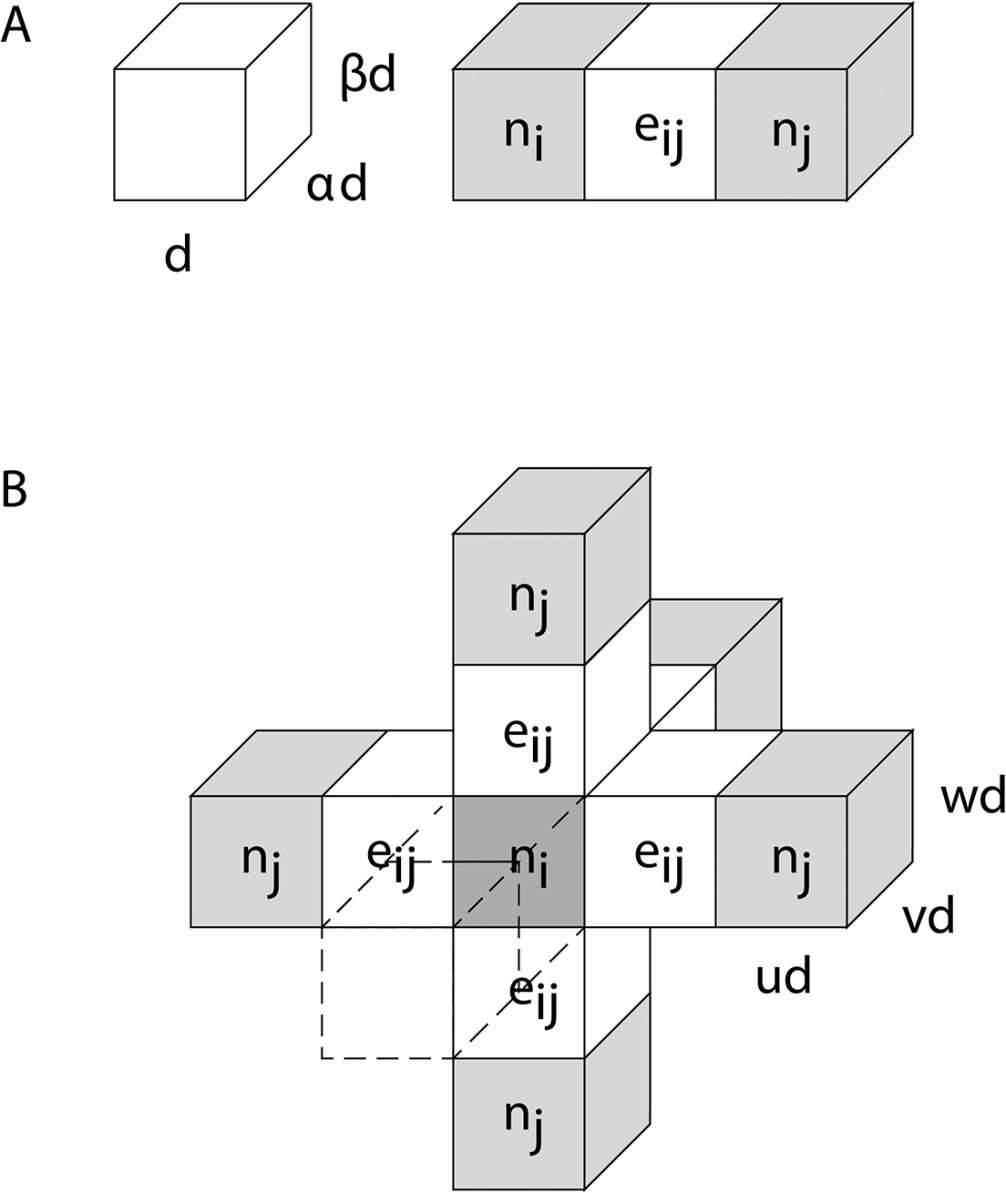
Three-dimensional edge, e_ij_ (white pixels), connecting nodes (gray pixels), n_i_ and n_j_, as shown in Parts A and B. As shown on the left in A, the voxel has dimension, *d* by *αd* by *βd*. Part B illustrates a fully connected 3D network with a central node, n_i_, (dark gray) connected to six other nodes (light gray), each through a single straight edge (white) similar to the fiber shown in Part A. The front node and edge are render transparent is this diagram to make the central node visible.

A network of simply connected nodes in three dimensions can be treated, using Eq 1, in a manner similar to the treatment of the two-dimensional graph above. However the edge weight for voxels that do not have an isotropic aspect ratio will depend on the aspect ratio of the voxels. For example, the edge weight shown in part A of Fig 4 between identical single-voxel nodes connected by an edge voxel in a straight line, is given by the following:

$$w(e_{ij})=\frac{1}{2}\left(\frac{(\alpha \beta )d^{3}}{(\alpha +\beta +\alpha \beta )d^{2}}\right)\frac{1}{d}\;,$$ (4)

where the *voxel-width* in Fig 4A is *d*, *αd*, or *βd*. The voxel has a volume of, (*αβd* ^*3*^, and a surface area of, *2αd* ^*2*^ *+2βd* ^*2*^ *+2αβd* ^*2*^ *= 2* (*α +β+αβ d* ^*2*^, so the edge weight depends on the orientation of the voxel. Eq 4 represents the edge weight for streamlines traversing left-right in Fig 4A, along the voxel-width *d*, hence providing an inverse length of 1/*d* (last term on the right-hand side of Eq 4). Therefore diffusion-weighted data should be acquired and processed with isotropic resolution to avoid an orientation bias in the calculation of streamlines and in the calculation of network parameters.

For isotropic voxels, Eq 4 will be simpler since the surface area, *A*_*voxel*_, of a single cubic voxel node is 6*d* ^2^ with a voxel volume, *V*_*voxel*_, of *d* ^3^. For the case of identical single, cubic-voxel nodes separated by *M* voxels in a straight line, the streamlines will have a length of *Md* and the number of streamlines will be determined by *MP*_*voxel*_, since each voxel will contribute to the fiber estimation. Therefore the edge weight defined in Eq 1 between these two single-voxel nodes will have a value of 1/6, since the resulting number of streamlines will be normalized by the number of seed-points-per-voxel and the number of voxels. Also using Eq 2, the connection strength will have a logical value of 1 for a fully connected network with a single node in the center of a network of identical single nodes on the type shown in part B of Fig 4 (were *u* = *v* = *w* = 1).

The edge weight and connection strength parameters can be used with more complex network graphs. For example, a rectangular node of many cubic voxels, with single-voxel width, *d*, that has “*u*” voxels in one dimension, “*v*” in the second, and “*w*” in the third dimension, has a surface area of that is the sum of the number of voxels in each of its six rectangular faces (“*uv*” will refer to the face of the node with surface area *uvd* ^2^, and so on for the other faces). The surface area of these nodes is *A* = (2*uv* + 2*uw* + 2*vw*) *d* ^2^. For any number of voxels in a straight edge of voxels connecting nodes with any number of seed points in each voxel, the number of streamlines in the face with cross section “*uv*” becomes *uvMP* and the length will be, *l* = *Md*. Using Eq 1, the edge weight is then given by

$$w(e_{uv})=\frac{d^{3}}{P}\frac{1}{(2uv+2vw+2uw)d^{2}}\sum_{p=1}^{P}\sum_{m=1}^{uvM}\frac{1}{Md}=\frac{uv}{2(uv+vw+uw)}$$ (5)

Therefore, the numerator in the sum depends on the surface area of the voxel face through which the edge connects the nodes, e.g. *uv*, however, if the edge were to connect through *uw* then the numerator would be *uw*. For a fully connected central rectangular node, as shown in part B of Fig 4, with an edge through each one of its faces (i.e., through *uv*, *vw*, and *uw* faces) that connects the central node to 6 other identical nodes, results in the following node connection strength:

$$s(n)=2w(e_{uv})+2w(e_{vw})+2w(e_{uw})=2\left(\frac{uv+vw+uw}{2(uv+vw+uw)}\right)=1.$$ (6)

The central node contains 6 edges, where the opposite face has the same edge (i.e. 2 edges through faces *uv*, 2 through *vw*, and 2 through *uw*). As for both the earlier two-dimensional and three-dimensional cases, the connection strength is equal to one for a rectangular node fully connected along a straight edge at each nodal face to identical rectangular nodes. In the case of rectangular nodes, the edges will have a different number of streamlines connecting each face, but changing the number of seed points per voxel and length of fiber (number of voxels in an edge) will not affect this result.

For connected nodes of isotropic voxels that are not identical, the value of edge weight and node connection strength will depend on the exact geometry of the connected nodes. To explore the range of possible edge weight values, consider the special case of two connected identical rectangular nodes that are connecting through an edge, which is one voxel thick (i.e. *w* = 1). The surface area of the nodes is *A* = 2*uvd* ^2^ + 2*ud* ^2^ + 2*vd* ^2^ and the edge length can be any arbitrary length, *l* = *Md*, where M is the number of voxels in the straight edge. The edge weight in Eq 5 is modified with *w* = 1 and is given by

$$w(e)=\frac{uv}{2(uv+v+u)}=\frac{uv}{2uv\left(1+\frac{1}{u}+\frac{1}{v}\right)}=\frac{1}{2\left(1+\frac{1}{u}+\frac{1}{v}\right)}.$$ (7)

For *u* = *v* = 1 (or *u* = *v* = *w*), *w(e)* will be equal to 1/6 (Eq 4 for isotropic voxels), but as *u* and *v* become very large, *w(e)* tends to an asymptotic value of 1/2. Therefore, the connection strength of any node will depend on the geometry of all the nodes in the connected network.

In summary, the edge weight presented here (Eq 1) provides a logical, dimensionless, scale-invariant measure of the connection strength between two nodes in a network. This edge weight is inversely proportional to the surface area of the connected nodes and directly proportional to the number of streamlines connecting the nodes. When the nodes are sufficiently defined by voxels smaller than the node size and the seeding is sufficiently dense, the edge weight provides a measure of the overall connectivity between two nodes relative to the surface area available to create connections. Also as shown above, the numerical value of the edge weight in an idealized, fully connected network provides a node connection-strength with unit value, which allows real networks to be directly compared to ideal networks of connected nodes. More complex geometries will be discussed in the next section using a combination of mathematical analysis and numerical simulations.

### 2.2. Numerical Analysis

To estimate network parameters in more realistic situations, two types of simulations were performed: 1) Edge weight was calculated for more complex fiber-pathway geometries using various seed densities, and 2) edge weight was calculated in the presence of random errors in the fiber direction estimation. All simulations were performed in IDL (Exelis Visual Information Systems, Boulder, CO).

**Fig 5:**
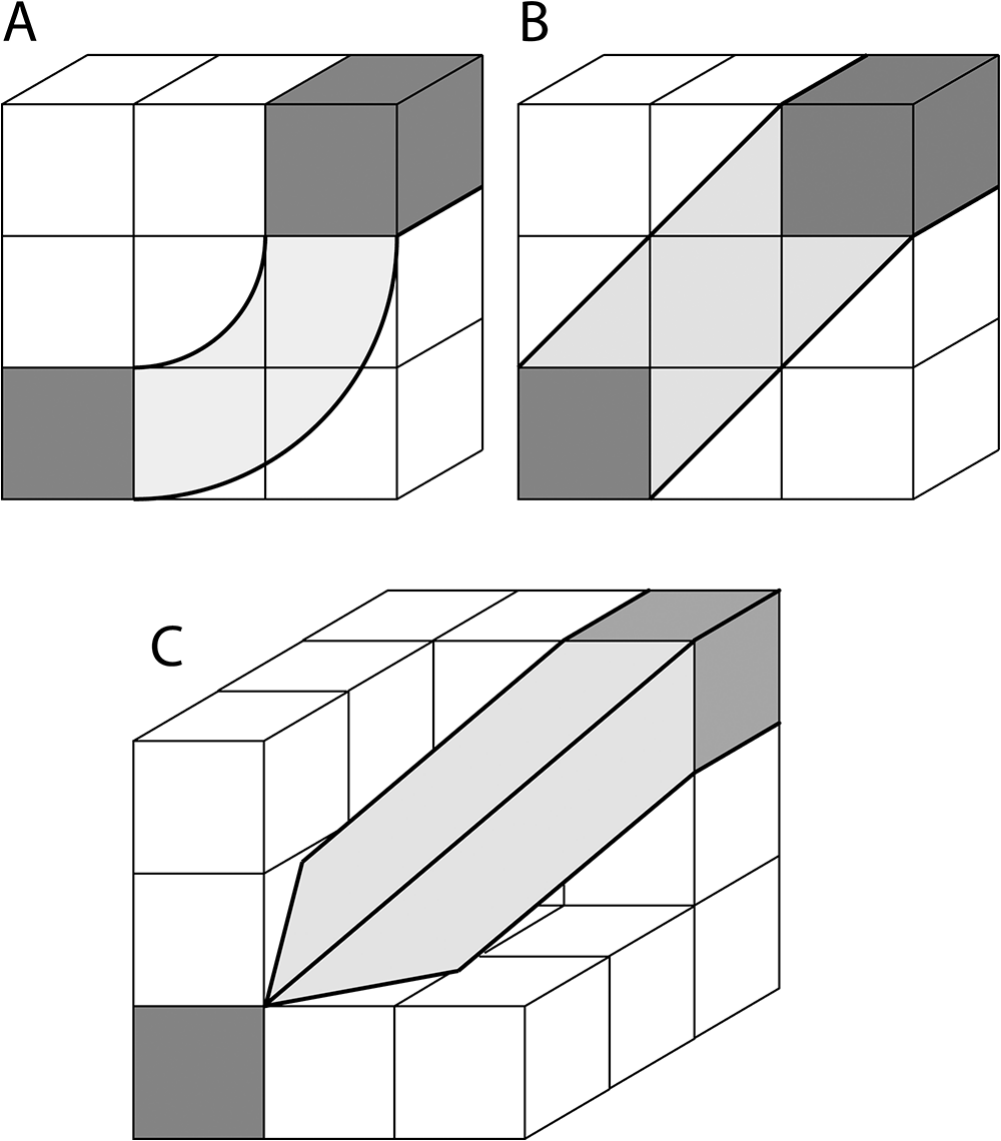
Diagram of two cubic, single-voxel nodes connected by a fiber in 3D. (A) Arched streamlines joining two nodes (dark gray) in the same plane of voxels connected at one face of each cubic node. (B) Nodes in the same plane of voxels connected by 45° streamlines at two adjoining orthogonal faces of each node. The nodes are separated by one or more voxels (one voxel separation shown in B). In this diagram, the streamlines joining the nodes at the closest point have a length equal to √2 times the voxel width. (C) Nodes connected at three adjoining orthogonal faces of each node by streamlines slanting at a polar angle of 45° and azimuthal angle of 54.1°. The nodes are in two voxel-planes separated by one or more voxels (one voxel separation shown in C). In this diagram, the streamline joining the nodes at the closet point will have a length equal to √3 times the voxel width.

Firstly to estimate the effect of increasing the seed density, the edge weight between connected single-voxel nodes was estimated for three edge geometries: 1) Through an arch of fibers (part A of Fig 5) in the plane of the nodes, 2) fibers slanted at 45° within the plane (part B) of the nodes, and 3) fibers slanted at a polar angle of 45° and an azimuthal angle of 54.1° (part C). The space between nodes was set to 1, 2, or 3 voxels, and the seed density was n^3^ and varied from n = 1 to 100. The seed points were placed uniformly in every voxel and across voxel boundaries. If the streamline originating from a seed point was a member of subset *R* in Eq 2 forming the edge between nodes, streamlines from that seed point were used to calculate the edge weight; otherwise, the streamlines were discarded. The results of these simulations can be directly compared to the analytical expression for the edge weight of the arched and slanted fibers presented in the Appendix.

Secondly to estimate the effect of noise on the edge weight calculation, where noise is characterized by a random Gaussian distribution in the estimated diffusion displacement orientation (e.g. the effect of noise on calculated diffusion directions), a simulation was performed for streamlines angled at 45° within a plane (part B of Fig 5) in a matrix of three-dimensional voxels. To estimate the effect of random error as the length of the streamlines connecting the nodes increase; the number of voxels between nodes was set to 1, 2, 3, 5 and 10 voxels. Without random errors, the streamline in each voxel was set to have unit displacement probability vector components, *v* = [0.707, 0.707, 0.0], in an x, y, z coordinate system. To simulate the effect of random errors, the diffusion displacement probability orientation in each voxel was modified by adding a random error to the orientation vector, with a standard deviation of σ set to 0 (no error), 0.03, 0.07, 0.1 or 0.2. For each level of random error, the streamlines were calculated with 5^3^ (125) seed points in each voxel with a tracking step size of half the voxel width. Then for each level of random noise, the calculation was repeated 105 times with a random assignment of orientation in each voxel.

### 2.3. MRI Acquisition

#### 2.3.1. Human brain *in vivo*

The University of Florida Institutional Review Board approved all human studies and consent forms. One healthy subject provided written consent to participate in this study and was scanned ten times over the course of one month, which provided a control set of ten acquisitions from which to estimate the variability of network properties across different MR acquisitions. With each scan, temperature and phantom based measurements were acquired to ensure consistency of scanner performance at each time point. The subject was scanned on a 3 T Siemens Verio system in the Shands Hospital of the University of Florida. High angular resolution diffusion imaging (HARDI) [37] data were obtained with a spin-echo prepared echo planar image [38] readout using the following set of parameters: TR/TE = 17300/81 ms, 2 scans without diffusion weighting, 6 diffusion gradient directions with b-values of 100 s/mm^2^ and 64 diffusion gradient directions with diffusion weighting 1000 s/mm^2^. The diffusion gradients were distributed following a scheme of electrostatic repulsion [39]. The diffusion-weighted images covered the entire brain with an isotropic resolution of 2.0 mm, field of view (FOV) of 256 mm x 256 mm, and 73 slices. This image was spatially interpolated to 1.0 mm isotropic resolution using cubic convolution [40] with the CONGRID function in IDL. In addition, a high-resolution T1 structural scan of the entire brain was acquired with TR/TE = 2500/3.77 ms, resolution 1mm isotropic, FOV of 256 mm x 256 mm and 176 slices.

#### 2.3.2. Excised rat brain

The rats perfused and placed in fixative at the University of South Florida (USF, St. Petersburg, FL) with the approval of the USF Institutional Animal Care and Use Committee (IACUC) permit #R3486. Four excised, fixed 90-day-old normal rat brains were examined with a repeated measurement on two brains for a total of six datasets. Prior to imaging, all brains were placed in buffered saline solution overnight to remove the free fixative. HARDI data was obtained using a 17.6 T Bruker Avance system (Bruker Corp, Billerica, MA) with the following set of parameters: 7 diffusion weightings 100 s/mm^2^ and 64 diffusion weightings of 2225 s/mm^2^. The diffusion gradients were distributed following the scheme of electrostatic repulsion. An image resolution of 190 x 190 x 190 μm^3^ was acquired; this dataset will be referred to throughout this text as the 190 μm dataset. Two new images were reconstructed for each of the six datasets. Interpolating the original image to 95 x 95 x 95 μm^3^ with cubic convolution yielded the second dataset, which will be referred to as 95 μm. Finally, degrading the original image by using only half of the original k-space information to reconstruct a new image yielded a resolution of 380 x 380 x 380 μm^3^, referred to as 380 μm dataset.

### 2.4. Data Processing

Using a rank-2 diffusion tensor model of diffusion, maps of FA and average diffusivity (AD) were created from the HARDI data using an in-house software written in IDL. The GM nodes were created by visual inspection using ITK-SNAP [41] (http://www.itksnap.org/) to delineate the desired structures on the FA and AD maps. For human data, the image segmentation was performed on a single dataset and registered to the other nine datasets using FSL’s FLIRT (http://fsl.fmrib.ox.ac.uk/fsl/fslwiki/FLIRT). Nodes were then registered using ApplyXFM by applying the matrix transformation from FLIRT. Each rat’s dataset was manually segmented using the defined structures in the Paxinos and Watson Rat Brain Atlas [42]. For rat data, the FA map of the 95 μm dataset was used for GM node segmentation. These structures were then registered onto the 190 and 380 μm images by applying an identity transformation using ApplyXFM. In this work, the method of Wishart distributed tensor [43] was used to characterize the displacement probability function in each voxel. This method allows the reconstruction of multiple displacement probability orientations in each voxel (i.e. resolves kissing or crossing fibers). The displacement probability function, streamlines, and edge weight (as defined in Eq 1) were calculated using an in-house software written in C. Deterministic tractography was performed, using a modified version of the fiber assignment by continuous tracking algorithm [44]. Streamlines were launched bilaterally (since diffusion is antipodal symmetric) and the direction of propagation was defined at each tracking step to be that which results in the least angular deviation from the direction defined by the previous streamline path step. Tractography was performed over a mask covering the entire brain using the following parameters: 125 seeds per voxel distributed within and across voxels, through voxels with FA larger than 0.05, a fiber step size of half the voxel size, and no step-to-step track deviations greater than 50°.

#### 2.4.1. Node Definition, Resolution, and Edge Weight Variability

The reliable segmentation of GM nodes, along with the definition of WM edges, is a crucial part of estimating connectivity in the brain. In MR images with appropriate contrast, the desired anatomical regions can be segmented as nodes by selecting voxels that meet the criteria of spatial location and contrast of the specific region. However limited spatial resolution (i.e., voxel size) may result in volume averaging, and ambiguity in the selection of the GM node boundaries and WM edge, that can affect the accuracy of the edge weight calculation. To investigate the effect of segmentation and resolution on network definition, edge weights and node connections strength were calculated for representative networks in human brains *in vivo* and in excised rat brains.

**Fig 6:**
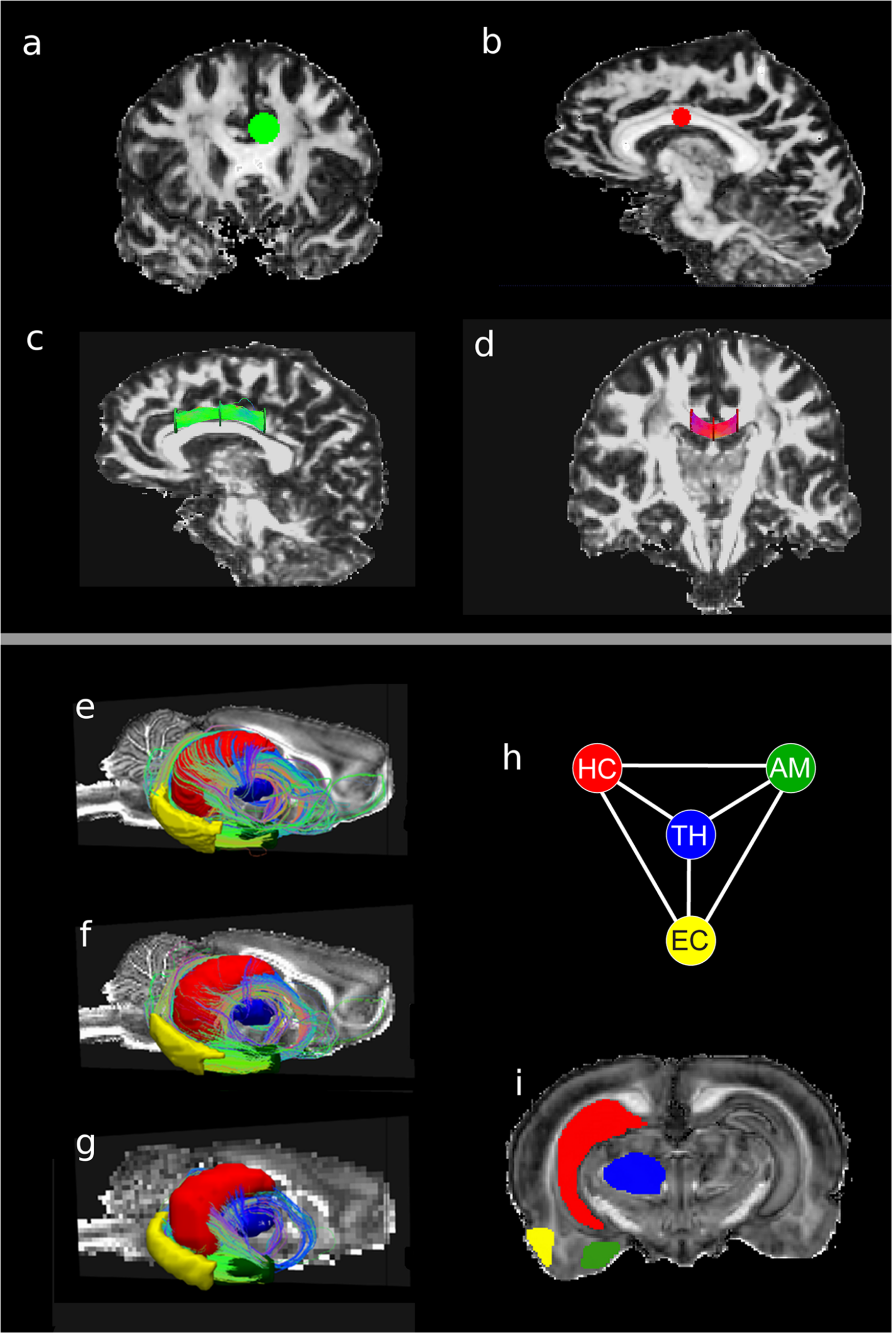
Human and rat brain networks. (A) Coronal view of a cingulum node. (B) Saggital view of corpus callosum (CC) nodes. (C) Saggital view of cingulum network shows disk nodes and streamlines connecting them. (D) Coronal view of CC network shows the disk nodes and fibers connecting them. Temporal lobe (TL) network with tracks connecting nodes (E) at the interpolated resolution of 95 μm, (F) at acquisition resolution of 190 μm, and (G) at the degraded resolution of 380 μm. (H) Sketch of the TL simple graph; hippocampus (HC), thalamus (TH), amygdala (AM), and entorhinal cortex, (EC). The color scheme is maintained in all figures. (I) Coronal slice displaying the TL rat nodes.

Firstly, several networks in the human brain were examined to determine the variability of streamline calculation and edge definition across the 10 data acquisitions on the same subject. As a first test, two regions of large, coherent WM fiber bundles in the cingulum and corpus callosum (CC) were selected, which are next to each other and the fibers travel orthogonally. Using FA images at 2 mm isotropic resolution, single voxel-thick, disk-shaped nodes with radii of about 6–7 mm were placed within the cingulum (surface area 459.2 mm^2^, see part A of Fig 6) and CC (surface area 309.2 mm^2^, see part B). Two-node and three-node networks were created in the cingulum, as shown in parts A and C. A long WM fiber bundle edge of roughly 49 mm length was defined between nodes 1 and 3 in the cingulum, as shown in part C. Placing a node in the middle of this long WM tract of the cingulum creates two cingulum short edges, between nodes 1 and 2 and between nodes 2 and 3 roughly 24 mm each. To study the edge weight estimation in the CC, nodes were placed laterally (22 mm apart) on each side of the body of the CC at the point where the WM starts to branch outward into the cortex, as shown in parts C and D. This edge will be referred to as the long edge of the CC, as shown in part D. A second three-node network is made by placing a third node at the midline of the body of the CC, creating two CC short edges of 10 and 11 mm.

Secondly, major structures in the temporal lobe (TL) in the excised rat brain were used to define a network of major nodes (i.e. only four nodes) by segmenting the brain using visual inspection from structures defined in the Paxinos and Watson Rat Brain Atlas [42]. This TL network was studied in four excised rat brains. A coronal view of the structures is shown in part I of Fig 6: the thalamus (TH) is blue, amygdala (AM) is green, entorhinal cortex (EC) is yellow and hippocampus (HC) is red. The same color scheme is used for the nodes in the TL network in parts E through H. To obtain a first estimation of the boundaries, the rat brain TL nodes were segmented using FA maps in ITK-SNAP from coronal slices using the Paxinos and Watson atlas. Further refinement was performed in the sagittal and transverse slices to achieve a 3D representation of the nodes with smooth contours. In segmenting the TH, the acoustic radiation, fasciculus retroflexus, and medial lemniscus were used as boundary markers. The HC included CA1, CA2, CA3 and dentate gyrus, but not the white matter structures of the alveus and fimbria, as well as the laterodorsal thalamic nucleus (used as a ventral boundary). The structure of the AM was kept separated from the white matter of the optic tract and a clear boundary was maintained between the AM and the piriform cortex. Since other dense anatomical structures are around and in contact the EC, the EC was defined to exclude the dorsal endopiriform nucleus and the piriform cortex. The subiculum was not included in order to physically maintain a separation between the EC and HC.

## Results

**Fig 7:**
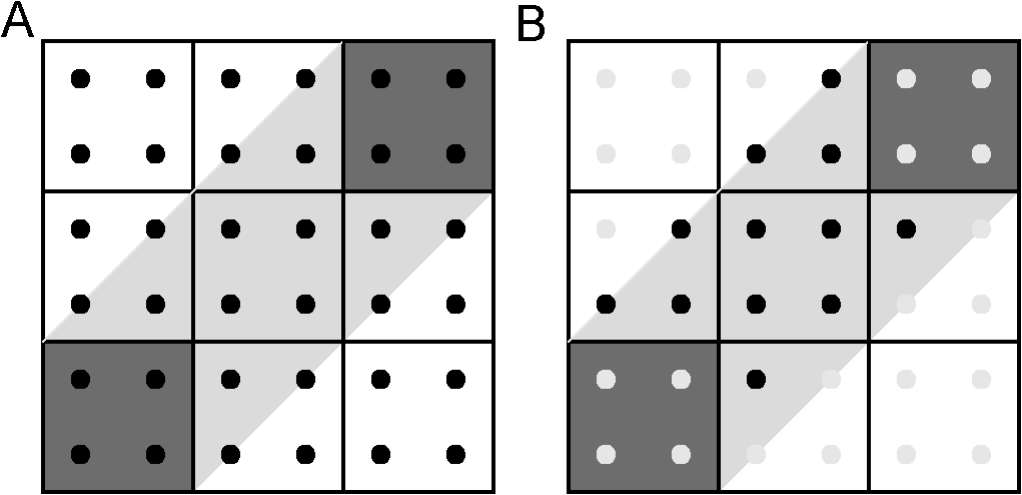
System of 2 nodes connected by a streamline at 45° with 4 equally spaced seed points in each pixel and across pixel boundaries. (A) Nodes (dark gray) are connected by a slanted fiber (light gray) and dark squares are the seeds points used to perform tractography. (B) After keeping the seed points that lie within the region, *R*, only 12 contribute to the edge weight out of 36 original seed points.

Since brain white matter fiber pathways are more complex than the simple examples discuss earlier (i.e. those in Figs 3 and 4), simulations were performed to estimate the edge weight in more complicated pathways, as shown in Fig 5. With this approach, the sensitivity of the edge weight and node strength calculations to geometry and data processing parameters were investigated. In complicated pathways, the location of seed points strongly influences the selection of the streamlines used in the edge weight calculation. For example, the effect of seed density location is shown in Fig 7, where not all 36 seed points from the set of 9 pixels contribute to the edge weight. As shown in part A, only 5 voxels contribute to the edge weight with a total of 20 possible seed points. Of the 20 possible seed points, only 12 contribute to the edge weight, as shown in part B. Therefore the effect on edge weight of seed point density and fiber path length in a voxelized image was investigated to estimate how placement of seed points within the discrete cubic geometry of voxels affects the calculation of the edge weight and node connection strength. Then using the results from these simulations as a guide, optimized methods were applied to edge weight calculations in large and coherent WM tracts in the 10 repeated DWI acquisitions of the same human subject. Also the connectivity between major structures in the rat brain temporal lobe network was estimated from the excised rat brain DWI data to investigate the dependence of network parameter calculations on changes in resolution and data interpolation.

### 3.1. Simulations

**Fig 8:**
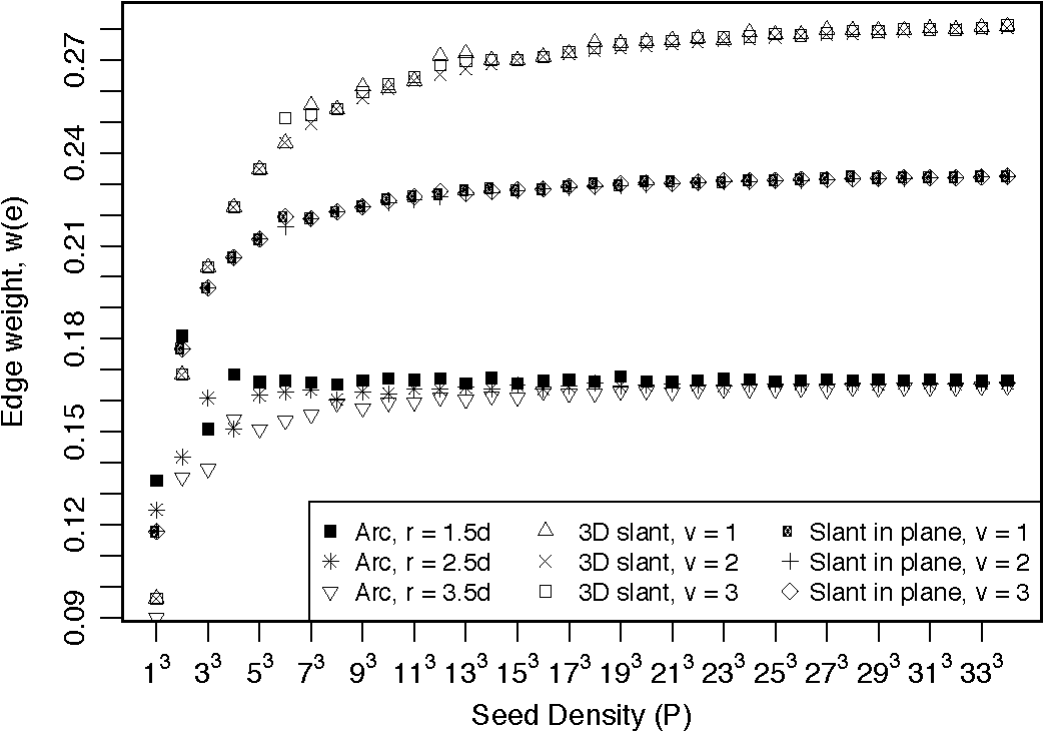
Edge weight calculations, for the arched and slanted streamline paths of Fig 4, as a function of seed point density. The seed point density is P = n^3^, where n = 1, 3, 5, …, 33. Arched edge weight values plateaus at a value of 0.167, for all radii, r (d is the voxel width). Slanted in-plane edge weight plateaus at a value of 0.235, and 3D slant edge weight plateaus at 0.289, when the nodes are separated by 1, 2, or 3 voxels (v).

Simulations were performed to study the effect of seed density and edge length (node separations) on the calculation of edge weights in ideal situations, with and without a noise contribution to the estimated primary diffusion direction. Without noise, the streamlines will not deviate as the path length increases. However as shown in Fig 8, the calculated edge weight value strongly depends on seed density and reaches a horizontal asymptotic value as the number of seeds per voxel increases, since this increase in seed density essentially allows more homogeneously sampling of actual streamline pathway defined by region *R*.

Simulations for the arched streamline (see part A of Fig 5) show this asymptotic behavior, when the nodes are separated by an arc with a radius of 1.5, 2.5, or 3.5 times the voxel width, as the seed density is increased to a high value (~ 200 or 6^3^ seed points per voxel) above which the edge weight reaches a plateau. The asymptotic values of 0.167 equals the expected values of 1/6 for identical cubic nodes fully connected along one face of each node (see the Appendix A.1). These results suggest that the edge weight values should be relatively independent of edge length, but the approach to an asymptotic value require a larger number of seed points to achieve stable edge weight. Edge weight results for the slanted fiber in-plane with the nodes (part B of Fig 5) reaches a plateau with values of 0.235, when the node separation is 1, 2, or 3 voxels, which is the expected value obtained from the analytical expression in the Appendix A.2. Similar to the arched fiber, these results suggest that a stable measure of connectivity requires a larger number of seed points. For this slanted fiber geometry, the streamline enters two faces of each node. Hence, the edge is covering a larger surface area of the nodes, but the streamlines occupy a larger volume, so the edge weight is proportionally larger. Edge weight results for the 3D slanted fiber (part C of Fig 5) reaches plateau values of 0.289, when the node separation is 1, 2, or 3 voxels, which is the expected value obtained from the analytical expression in the Appendix A.3. As these simulations imply, a larger number of seed points are needed to obtain a stable measure.

As the geometry of the fiber path becomes more complex, the number of seed points must increase to accurately calculate the edge weight for these pathways. In all simulated fibers, a large seed density is required to reach the plateau in the edge weight value (P > 8000 for value within 1% of the plateau value for each case). But increasing the number of seeds per voxel dramatically increases computation time and data storage requirements. Cheng et al. [29] suggest that high seed densities reduce variation in calculated brain network parameters, similar to the results presented here. However, the highest seed density employed in their work was *P* = 40 [29]. The results presented here for the edge weight of Eq 1 suggest that 40 seed points are not sufficient to provide an acceptable estimate of the edge weight between nodes. For these simulations, using a seed density of 125 results in a calculated edge weight that is approximately equal to the expected value for straight or arched streamlines connecting a single face of each node, while only ~ 90% of the expected value for streamlines slanted in the plane of the nodes, and ~ 80% of the expected value for streamlines slanted in all three dimensions. However an entire brain tractography file with *P* = 125 yielded a file size for human data of ~ 250 GB and rat data ~ 500 GB. The larger file size of the tractography in the rat data is due to the high spatial resolution obtained in these datasets. These results suggest that *P* = 125 is an appropriate compromise between storage capacity and high seed densities that will provide reasonable results. Therefore a seed density of 125 is used is this study, but the results might be improved with higher seed density when appropriate computation resources are available.

**Fig 9:**
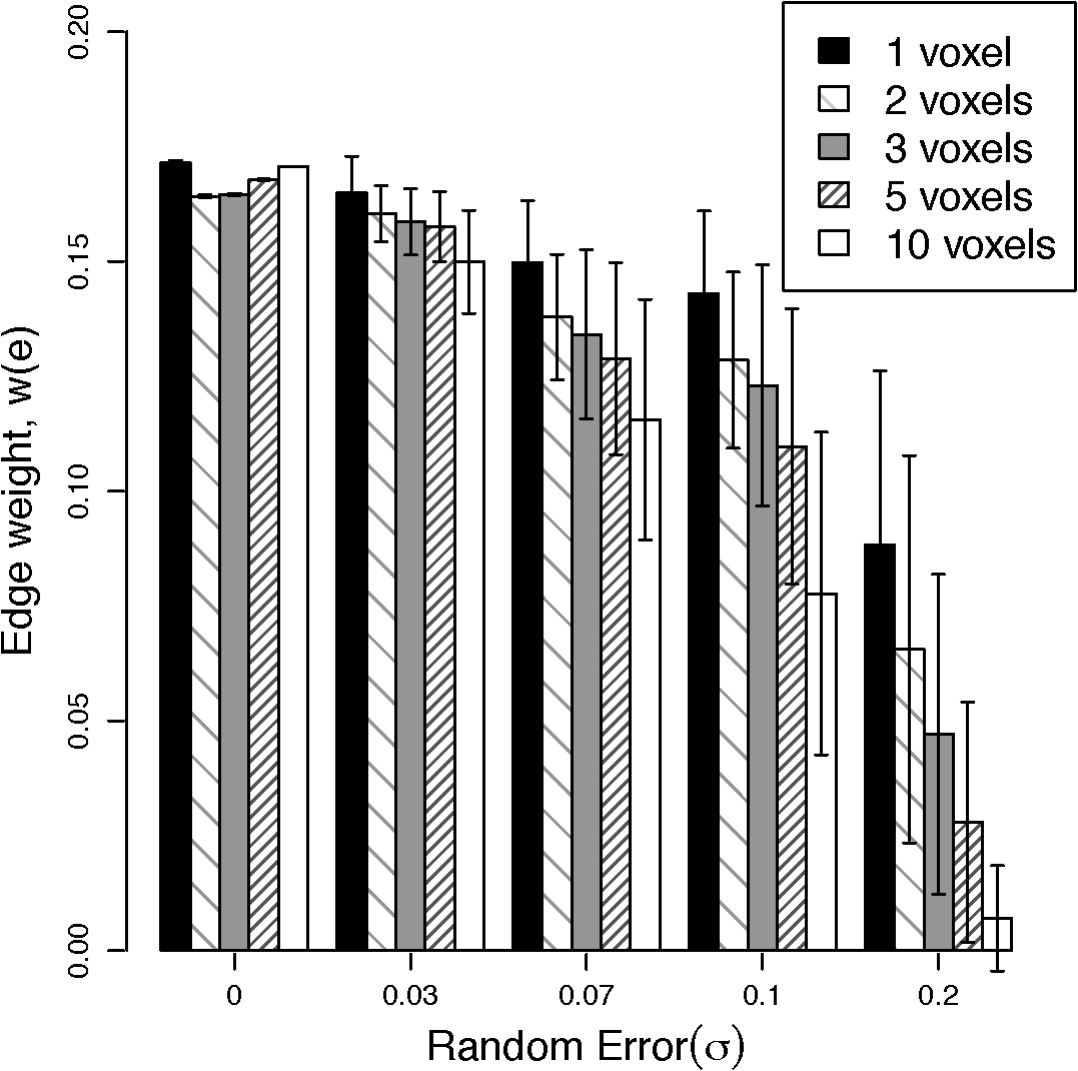
Simulation of edge weight values for an arc streamline pathway in the same plane as single voxel nodes (Fig 5, part A) as random error increases. The nodes closest points were separated by 1, 2, 3, 5, and 10 voxels.

To simulate the effects of noise in the estimated diffusion directions, the edge weight between identical nodes for slanted fibers, in-plane with the nodes (part B of Fig 5), was calculated as the standard deviation, σ, in the diffusion direction at each step increased. With *P* = 125, the simulated edge weight value increasingly deviated from the expected value (1/6 for these slanted streamline) as the edge length increased and the noise increased. Since the edge weight is derived from tractography, which in turn is derived from DWI measurements, appropriate levels of SNR are needed to estimate connectivity. Bastin et al. [45] showed that SNR of 20 or higher is necessary to obtain stable tensor measures from DWI. The results illustrated in Fig 9 indicate that noise levels below σ = 0.03 will result in edge weight values close to the expected value (differences of 7.5% or less) and suggest that measurements with SNR greater than 30 are needed to obtain a stable estimate of edge weight.

The formulation of the edge weight presented here is normalized to remove the dependence on seed density and to be size-scale invariant. Therefore a sufficiently high seed density should be selected to assure that the edge weight is independent of seed density. However the edge weight is only independent of size scale if the WM fibers of interest are sufficiently resolved. At a spatial resolution that is low relative to the WM fibers of interest, small fiber pathways may not be resolved sufficiently to allow the calculation of streamlines, even at extremely high seed densities, due to volume averaging with other pathways and surrounding tissue. When a WM pathway is not sufficiently resolved, increasing the seed density will only replicate the same streamlines and may increase the number of false positives [46]. Therefore to quantify the edge weight, streamline tracking requires sufficient spatial resolution and an appropriate seed density, which necessitates a compromise between image measurement time and computational requirements.

### 3.2. Human Brain Cingulum and Corpus Callosum Networks

The edge weight was calculated between disk nodes placed in the cingulum and CC WM tracts in ten datasets from a single human subject. These tracts were selected because they represent large, coherent, and fairly homogenous WM structures in the brain. Also the repeated acquisitions from a single subject provide a good assessment of the variation in edge weight determination for large WM tracks across image acquisitions from the same individual. For all 10 datasets, the acquisition parameters were identical and the SNR was constant (~ 52 at b = 100 s/mm^2^, and ~ 26 at b = 1000 s/mm^2^). Table 2 shows the calculated average edge weight in these two major WH regions from the 10 repeated measurements of the human subject and the coefficient of variation with and without spatial interpolation, and with and without streamline restriction (i.e. with and without the indicator function). To examine the simplest form of resolution enhancement, the diffusion-weighted images were spatially interpolated, with cubic convolution, by a factor of 2 in each dimension before the calculation of displacement probability. Although vector field interpolation of the displacement probability function [47] may produce optimal streamlines, the overall results in Table 2 indicate that the calculated edge weight has lower variability with streamline restriction which is additional improved with cubic convolution interpolation of the diffusion weighted images.

**Table 2:** Edge weight, *w*(*e*), for edges, *e*, in major white matter regions (CC, corpus callosum; Cing, cingulum). Calculated average *w*(*e*) value from the ten human datasets at 1 and 8 mm^3^ isotropic resolution, along with the associated coefficient of variation, *c_v_*, of the edge weight. The results are presented for the complete edge, CC long and Cing long, as well as two subdivisions (short 1 and short 2, see text) of these long edges (see Fig 6). The calculations were performed without restriction to the streamlines connecting the nodes (Without Streamline Restriction), and with the inclusion of streamlines fibers restricted (With Streamline Restriction) to originate from the connecting edge, *R*.

*Edge* (*e*)	*Without Streamline Restriction*				*With Streamline Restriction to R*			
	*1 mm* ^*3*^		*8 mm* ^*3*^		*1 mm* ^*3*^		*8 mm* ^*3*^	
	*w*(*e*)	*c*_*v*_ (*%*)	*w*(*e*)	*c*_*v*_ (*%*)	*w*(*e*)	*c*_*v*_ (*%*)	*w*(*e*)	*c*_*v*_ (*%*)
CC long	9.42E-01	10.44	6.06E-01	15.24	1.33E-01	7.57	9.62E-02	11.13
CC short 1	2.26E+00	13.09	1.62E+00	15.01	1.56E-01	9.29	1.16E-01	9.55
CC short 2	2.77E+00	9.00	1.95E+00	13.29	1.79E-01	8.54	1.33E-01	9.88
Cing long	2.56E-01	12.36	1.50E-01	15.12	6.12E-02	9.52	4.31E-02	12.49
Cing short 1	5.64E-01	9.76	3.57E-01	11.86	7.66E-02	7.74	5.75E-02	10.73
Cing short 2	7.02E-01	9.80	4.06E-01	13.57	8.87E-02	7.80	6.26E-02	8.15

The percentage difference between edge weights of longer edges compared to those of shorter edges was calculated to observe discrepancies in the edge weight as a result of node placement. Since the edge weight is a measure of connectivity strength, the edge weight of the longer and shorter edges should only vary due to the seed point effects, as long as the streamline is completely contained within the edge between the nodes. The proposed method of streamline restriction showed a difference between long and short CC edges in the 1 mm data of 16.1% and 30.0%, and the 2 mm data showed 18.7% and 32.2% difference. But including all streamlines without restriction, the percent difference in edge weight of the long to short edges was found to be much greater (82.2% and 98.7% for the 1 mm datasets and 91.4% and 105.1% for the for the 2 mm datasets). The same analysis was performed on the cingulum tract. The percentage difference in edge weight with streamline restriction between the longer and shorter edges was found to be 22.3% and 36.7% in the 1 mm data, and 28.7% and 37.0% in the 2 mm data. Without restriction, the percent difference in edge weight of the longer edge relative to the shorter edges was found the be 75.2% and 93.2% for the 1 mm data and 81.4% and 92.0% for the 2 mm data. Therefore, the filtering of streamlines should ensures that only streamlines originating from within the pathway are used when estimating connectivity by eliminating streamlines that originate from outside the pathway (voxels outside of region *R*), which might otherwise contribute to the edge weight calculation.

### 3.3. Rat Brain Temporal Lobe Network

**Fig 10:**
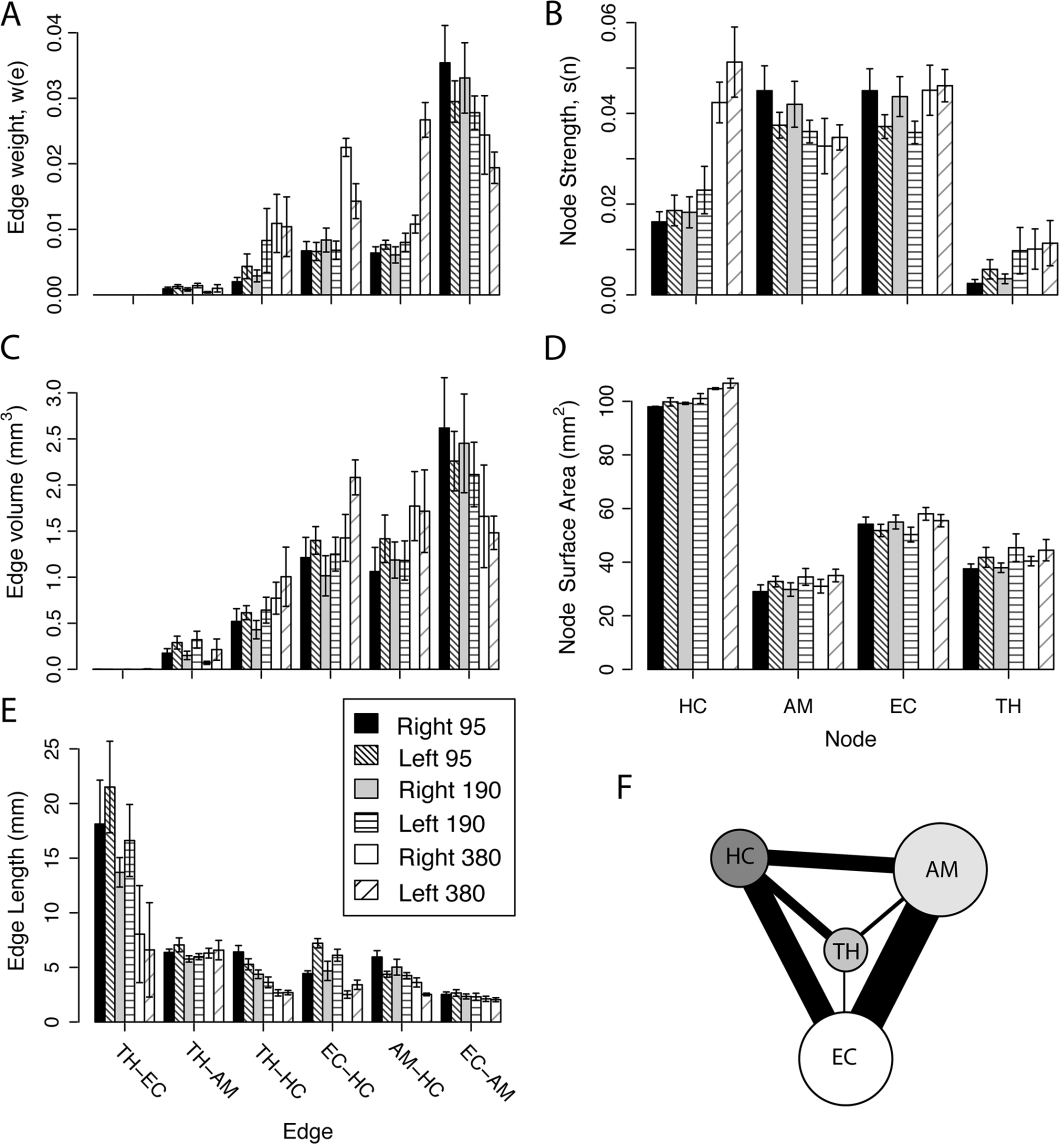
Excised rat brain TL network parameter values at isotropic resolutions of 95, 190 and 380 μm. The edge values are in the left column and node values in the right column: (A) Edge weights values (TH-EC values are too small to appear at this scale), (B) node strength values, (C) edge volume, (D) node surface area, and (E) edge lengths. (F) The highest resolution TL network with the edge widths size scaled by the value of edge weight and the node size scaled by the value of the node connection strength. The TH-EC edge is illustrated at the minimum line width that is still visible.

The major structures in the rat TL network are shown in Fig 6, part E through G, at three different spatial resolutions. The data was acquired with an isotropic resolution of 190 μm (part F) in 20 hours with acquisition parameters that represent a compromise between SNR, spatial resolution, and the DWI parameters. In the highest spatial resolution dataset (part E), the TL network displays a set of long streamlines wrapping around the HC (red node), while the lower spatial resolution dataset shows much fewer streamlines connecting to the surface area of the HC (part G). The edge weights are shown in part A of Fig 10 for the six edges in the left and right side TL network at 95, 190 and 380 μm isotropic resolution.

As discussed in the development Eq A-2 of the Appendix, the edge weight defined in Eq 1 can be conceptualized as the ratio of the edge volume divided by the total edge volume available (product of the nodal surface-area average and the edge length). Here the edge volume is the product of voxel volume and the number of streamlines in the edge, since each voxel in an edge will contribute a streamline to the total number of streamline (see Eqs A-1 and A-2). The extremes for the concept of volume ratios described in Eq A-2 are shown by the following: The TH-EC edge has a very low weight (on the order of 10^−6^), since the edge volume is smaller (see part C of Fig 10) and the edge length is longer (see part E) than the other edges in the TL network. In three of the six rat brains at the lowest resolution of 380 μm, no streamlines in the TH-EC edge met the criteria discussed in the edge weight section. So the TH-EC edge weight was assigned a value of zero for these three brains at 380 μm resolution. At the other extreme, the EC-AM has the highest edge weight, since the edge volume is larger and the edge length is shorter that than the other nodes. As indicated by the simulations, longer streamlines increasingly become more difficult to estimate accurately, due to error propagation in the tractography process. The edge weight variation (error bars) for any particular edge was similar at all resolutions, but the weights of edges to the HC consistently increases with decreasing resolution in the left and right side networks, whereas the EC-AM edge weight decreases with decreasing resolution, which is a reflection of the resolution dependence of the edge volume and edge length.

As shown in part D, the nodal surface areas are relatively independent of resolution and their variability is similar. Therefore surface area resolution dependence does not contribute much to the resolution dependence of the edge weight. In addition, the resolution dependence is minimized because the nodes were segmented using the high-resolution images as a reference. The HC is the largest and most consistently defined node with surface area variations of less than 3% at all resolutions. The AM, EC and TH are in close spatial proximity to other anatomical structures with similar contrast, which made the segmentation of these nodes more difficult than the HC. Ample care was taken to consistently define the TL nodes, which resulted in the small variation in the surface areas observed.

The node strength (see part B of Fig 10) shows similar results for left and right TL networks and the low variability suggests some robustness in this metric. But the node strengths of the HC and TH increase with decreasing resolution, the AM decreases slightly with decreasing resolution, and the EC did not show much resolution dependence. The HC and TH are central nodes connected to the other nodes through longer WM pathways. As the spatial resolution decreases, the calculated length of the edges connected to the HC and TH nodes decrease and the edge volume increases to yield larger edge weights as the resolution decreases, which results in an increase in the connection strength of these nodes as the resolution decreases. The AM node is located in an inferior part of the TL network (Fig 6) and only connects to other TL structure through shorter pathways. Lower spatial resolution minimizes the number of voxels that contribute to the weight of edges connected to the AM, so the estimation of these streamlines becomes increasingly difficult without adequate spatial resolution. Finally the EC is situated such that pathways connecting the EC to the HC are enhanced at low spatial resolution. While at high spatial resolution, the small pathways connecting the EC to the TH and AM become better characterized, hence making the EC node strength consistent across changes in spatial resolution. Therefore in the rat brain, resolution higher than 380 μm is needed to estimate the connectivity in the TL network. These results support the notion that spatial resolution is a limiting factor in resolving edges between nodes.

## Discussion

Using streamline tractography, large-scale fibrous brain structures have been studied *in vivo* as binary networks [15–21]. While binary connections provide insight into network structure, weighted networks are better suited to the study of the connection strength between nodes in brain networks, particularly smaller local networks, such as the TL. The connectivity of the TL network corresponds to a completely connected network, as shown in Fig 6. Employing a binary characterization of this network no new information is obtained, since these nodes are known to be connected. On the other hand, weighted networks add another degree of freedom in the characterization of these local networks and create a more realistic representation of the brain.

As shown in Fig 8, a high seed density is required to reduce the variation in the calculated connectivity metrics, which is consistent with previous reports [29]. However, with our computing resources, a compromise was necessary between computational requirements (computational time and data storage) and seed density, to reliably map the streamline connectivity. The simulations suggest that a seed density of several thousand would be optimal, but this would require excessive computation resources beyond the capacity available for this study. Therefore a seed density of 125 is used as a compromise, between the limitations of computational resources and the optimal seed density, to minimize the effect to roughly 10% or less of expected values in simulations, but avoids excessively large tractography files. Some of the computational and storage limitations could be overcome with novel tractography techniques [48] that take advantage of the increased computing power. In addition, innovative tractography algorithms can be used to improve tracking of streamlines used to calculate the edge weight [49, 50]. The need for large seed densities has been suggested [29] independent of weighting scheme, but this study is the first time a proposed edge weight has been developed to address the needs of a higher seed density while maintaining a convergent characteristic as seed density is increased, and the scale changed.

In Fig 9, the results of the simulations show an increased uncertainty in the edge weight value as random errors in the estimated diffusion direction and the node separation increase, as previous reported for fiber tracking [45, 51]. For σ levels less than 0.03, the percentage difference in the edge weight between the shortest and the longest streamlines are less than 11.9% and the *c*_*v*_ less than 7.5. Therefore, high SNR in the DWI acquisitions is needed to maintain accurate fiber estimation [52] and to allow the calculation of a reproducible edge weight measure.

Using the indicator function to restrict the inclusion of streamlines (see Eq 2), the calculated corpus callosum and cingulum edge weights are more consistent. Since the number of white matter fibers should not vary greatly along the length of either structure, so the edge weights for the long and short edges should be similar. As shown in Table 2, the difference between the long and short edges and the coefficient of variability is greatly reduced with streamline restriction. But edge weight does not depend solely on the number of streamlines in the edge. For an edge of constant cross section, the number of fibers is proportional to the cross sectional area of the edge. For example in the 8 mm^3^ resolution data, the cross sectional area of the streamline path between the nodes in the cingulum is approximately 58.4 mm^2^ and for CC is 87.2 mm^2^, but the long cingulum edge weight is only 55% of the long CC edge weight. This reflects the fact that cingulum nodes have roughly 48.5% larger surface area (459.2 mm^2^) than the CC nodes (309.2 mm^2^) and the edge weight is inversely proposal to the average surface area of the connected nodes. This illustrates that the edge weight depends strongly on the nodal surface areas (size of the nodes) as well on the number of streamlines connecting the nodes.

For the excised rat brain network (shown in parts E through I of Fig 6), streamlines connect all four nodes in this four-node TL network and are mainly concentrated near the HC (red ROI). Segmentation of nodes (see part I of Fig 6) at the lowest resolution was not possible because no clear node boundaries could be identified. Therefore node segmentation was performed using the high-resolution images, and then nodes in the lower resolution images were derived by registering high-resolution image nodes to the low-resolution images. This node segmentation procedure results in low variability of surface area estimates for all spatial resolutions (see part D of Fig 10). However, there is a slight increase in node surface area as resolution decreases.

At lower resolution, white matter and gray matter may average at the boundary of a node, so that fewer voxels will yield coherent pathways. As shown in part E of Fig 10, TH-EC edge is the longest and has the highest variability, while the variation is much less in the other shorter edges, which is consistent with the findings of Miles and Laidlaw [51], who noted that the effect of noise on calculated streamline tracts increases with increasing edge length. Also the TH-EC edge proved difficult to resolve given its small size and long length.

As shown in part C of Fig 10, the number of streamlines in the edge (edge volume) is strongly dependent on resolution. In this figure, the dependence of the number of streamlines on seed density is removed by dividing all values by the seed density (of 125 in this case). Then the direct dependence on resolution is removed by calculating the edge volume. In part A of Fig 10, the edge weight is also strongly dependent on resolution with the most significant dependence exhibited by edges connected to the HC. Thus the node strength, shown in part B of Fig 10, more strongly depends on resolution for the edges to the HC. Because the EC and AM nodes are close, the edge between them has the shortest length (see part E of Fig 10), but has the highest edge volume and weight; thus parts E through G of Fig 6 display a coherent fiber structure connecting the EC and AM nodes. Assuming the result calculated from the highest resolution image data provides the most accurate results, a final diagram representing the four-node network is shown in a Part F of Fig 10. For this figure, the edge width is scaled by the edge weight and the node size by the node connection strength. The TH-EC edge is illustrated at the minimum line width that is still visible in the diagram.

## Conclusion

Previously brain connectivity studies mostly used binary network descriptions of the cortex [15–17], but weighted networks provide a more natural description of brain network connectivity [23, 25, 26]. The dimensionless and scale-invariant formulation of the edge weight in Eq 1 removes the direct influence of seed density and spatial resolution on the estimation of network characteristics, like edge weight and node strength, which diverge as seed densities increase for other formulations of edge weight (Fig 1). Therefore, the use of the edge weight, defined in Eq 1, clearly demonstrates the need for high seed densities to increase network metrics reliability [29]. An appropriate SNR level and sufficient resolution are essential for the acquisition of optimal data to estimate the fiber paths connecting anatomical structures [45, 51], particularly for subcortical to cortical connections. Errors associated with low SNR affects the estimated diffusion profiles leading to error propagation in streamline tractography, thus reducing confidence that the result represents actual long fiber pathways. But even with higher SNR, improvements in the spatial resolution of diffusion profiles [11], along with optimized interpolation schemes [47, 53], may improve streamline tractography.

Seed density and volume averaging effects are more important at spatial resolution low relative to the actual WM tract size, suggesting that high seed densities and higher resolution will reduce the variability of weighted network metrics. The selection of a high seed density requires a compromise between accuracy and computation resources, but this may be overcome as available computation resources improve. However, higher spatial resolution requires a compromise between time and SNR. Therefore the ability to reliably quantify streamline tracts depend on having sufficient SNR with appropriate spatial and angular resolution, which will ultimately allow the use of tractography to estimate fiber pathways and the creation of weighted networks in the brain.

## Appendix: Analytical Expression for Edge Weight

In this appendix, an analytical expression is developed for the edge weight of Eq 1 in order provide a prospective on the value of edge weight expected in the idealized geometries shown in Fig 5. Starting from Eq 1, the following rearrangement of terms,

$$w(e_{ij})=\left(\frac{1}{P_{voxel}}\right)\left(\frac{2}{A_{i}+A_{j}}\right)\sum_{p=1}^{P}\sum_{m=1}^{M}\frac{\chi_{R}(f_{p,m})V_{voxel}}{l(f_{p,m})}\;,$$ (A-1)

suggests an analytical expression for the edge weight of the following form,

$$w(e_{ij})=\frac{1}{\langle A\rangle }\frac{1}{\bar{l}\left(f_{R}\right)}\int_{R}\;dV\;,$$ (A-2)

where the integral over *R* defines the volume occupied by the streamlines defined by the indicator function, ⟨*A*⟩ is the mean surface area of the nodes, and $\bar{l}\left(f_{R}\right)$ is the average length of the streamlines. Explicitly ⟨*A*⟩ is given by,

$$\langle A\rangle =\frac{\int \;\int_{T}\left(\sqrt{{\left(\frac{\partial g_{1}}{\partial x}\right)}^{2}+{\left(\frac{\partial g_{1}}{\partial x}\right)}^{2}+1}\right)dx\;dy+\int \;\int_{T}\left(\sqrt{{\left(\frac{\partial g_{2}}{\partial x}\right)}^{2}+{\left(\frac{\partial g_{2}}{\partial x}\right)}^{2}+1}\right)dx\;dy}{2}\;,$$ (A-3)

where *g*_*i*_ is the function describing the surface along the *z* axis of node *i* and *T* is the region of the node occupied in the xy plane. The mean length of the streamlines in the edge located within region *R* is specified by

$$\bar{l}(f_{R})=\frac{1}{s_{2}-s_{1}}\int_{s_{1}}^{s_{2}}s\;dl=\frac{1}{s_{2}-s_{1}}\int_{s_{1}}^{s_{2}}\left(\int_{0}^{l}\sqrt{1+f\prime {\left(x'\right)}^{2}}\;dx'\right)dl\;,$$ (A-4)

where

$$s=\int_{0}^{l}\sqrt{1+f\prime {\left(x\prime \right)}^{2}}\;dx\prime \;,$$ (A-5)

The function, *f*(*x*), is the trajectory of the fiber with slope *f’*, and *s* is the length of individual fibers.

In the denominator of Eq A-2, the volume measure, $\langle A\rangle \bar{l}\left(f_{R}\right)$, can be considered topologically to represent the total volume available for connection between the nodes, analogous to the total volume of a cylinder (i.e. cross section area times length), since the surface of the nodes can be flattened to represent the cylinder base and the mean fiber length to represent the cylinder height. Since the numerator is the volume occupied by the streamlines in the edge connecting the nodes, the edge weight represents a ratio of the edge volume occupied by the streamlines to the total volume available for streamline connections between the nodes. From this perspective, the edge weight can be seen to be independent of streamline length.

### A.1. Calculation of the edge weight for an in-plane straight or arced edge

Using the concept that the edge weight is the ratio of volume occupied to volume available, the edge weight value between equivalent cubic nodes, joined along one face of each node, should be 1/6 independent of the streamline path, as illustrated in the examples shown in Figs 3 and 5A.

### A.2. Calculation of the edge weight for an in-plane slant edge

The edge weight expression in Eq A-2 can be used to calculate the strength of connectivity between the two nodes shown in Fig 5B. The surface area of a cubic node with sides *d* is given by

$$\langle A\rangle =6d^{2}.$$ (A-6)

The average fiber length, with any number of voxels separating the nodes is given by

$$\begin{array}{l} \bar{l}=\frac{1}{M+1-M}\int_{Md}^{(M+1)d}\;\int_{0}^{l}\sqrt{1+1}\;dx\prime dl=\sqrt{2}\int_{Md}^{(M+1)d}{x\prime |}_{0}^{l}dl=\sqrt{2}d{\frac{l^{2}}{2}|}_{M}^{M+1}\\ =\sqrt{2}d\frac{{\left(M+1\right)}^{2}-M^{2}}{2}=\sqrt{2}d{\frac{2M+1}{2}}_{.} \end{array}$$ (A-7)

Alternatively, the average length is the average of the shortest and longest fiber. In this case, all fibers are distributed evenly in the xy plane, forming a square cross section of fibers connecting the nodes. The mean fiber length obtained by

$$\bar{l}=\frac{\sqrt{2}Md+\sqrt{2}(M+1)d}{2}=\sqrt{2}d\frac{2M+1}{2}\;,$$ (A-8)

which is the same result of Eq A-7. For this case, the volume occupied by the fiber in the region *R* (volume integral in Eq A-2) is obtained by

$$\int_{R}\;dV=\{\begin{array}{c} R_{1}\;0<z<d,\;0<x<d,\;0<y<d(x+1)\\ R_{2}\;0<z<d,\;d<x<d(M+1),\;dx<y<d(x+1)\\ R_{3}=R_{1}+R_{2}. \end{array}\;.$$ (A-9)

First, the integral over *R*_*1*_ is given by

$$\int_{R_{1}}dV=\int_{0}^{d}\left(\int_{d}^{(x+1)d}\left(\int_{0}^{d}dz\right)dy\right)dx=d\int_{0}^{d}{y|}_{d}^{(x+1)d}dx=d^{2}\int_{0}^{d}x\;dx=d^{3}{\frac{x^{2}}{2}|}_{0}^{1}=\frac{d^{3}}{2}.$$ (A-10)

Then, the integral over *R*_*2*_ is obtained by

$$\int_{R_{2}}\;dV=\int_{d}^{(M+1)d}\left(\int_{xd}^{(x+1)d}\left(\int_{0}^{d}dz\right)dy\right)dx=d^{2}\int_{d}^{(M+1)d}1\;dx=d^{2}\int_{d}^{(M+1)d}dx=d^{3}\;{x|}_{1}^{M+1}=d^{3}M.$$ (A-11)

Finally, employing the expression for the entire volume *R* (Eq A-9) and the solutions of Eqs A-10 and A-11 yields the volume for the entire region *R* by the following expression,

$$\int_{R}\;dV=2\left[\int_{R_{1}}dV+\int_{R_{2}}dV\right]=2d^{3}\left[M+\frac{1}{2}\right]=d^{3}(2M+1).$$ (A-12)

In conclusion, the edge for the slant on a plane where the nodes’ surface area is specified by Eq A-6, a mean edge length equal to Eq A-8 and the volume occupied by the fiber obtained by Eq A-12, yields an edge weight given by

$$w(e)=\frac{1}{6d^{2}}\frac{1}{\sqrt{2}d\frac{\left(2M+1\right)}{2}}(2M+1)d^{3}=\frac{1}{3\sqrt{2}}\approx 0.235,$$ (A-13)

regardless of the physical length of the edge.

### A.3. Calculation of the 3d-slanted edge

**Fig 11:**
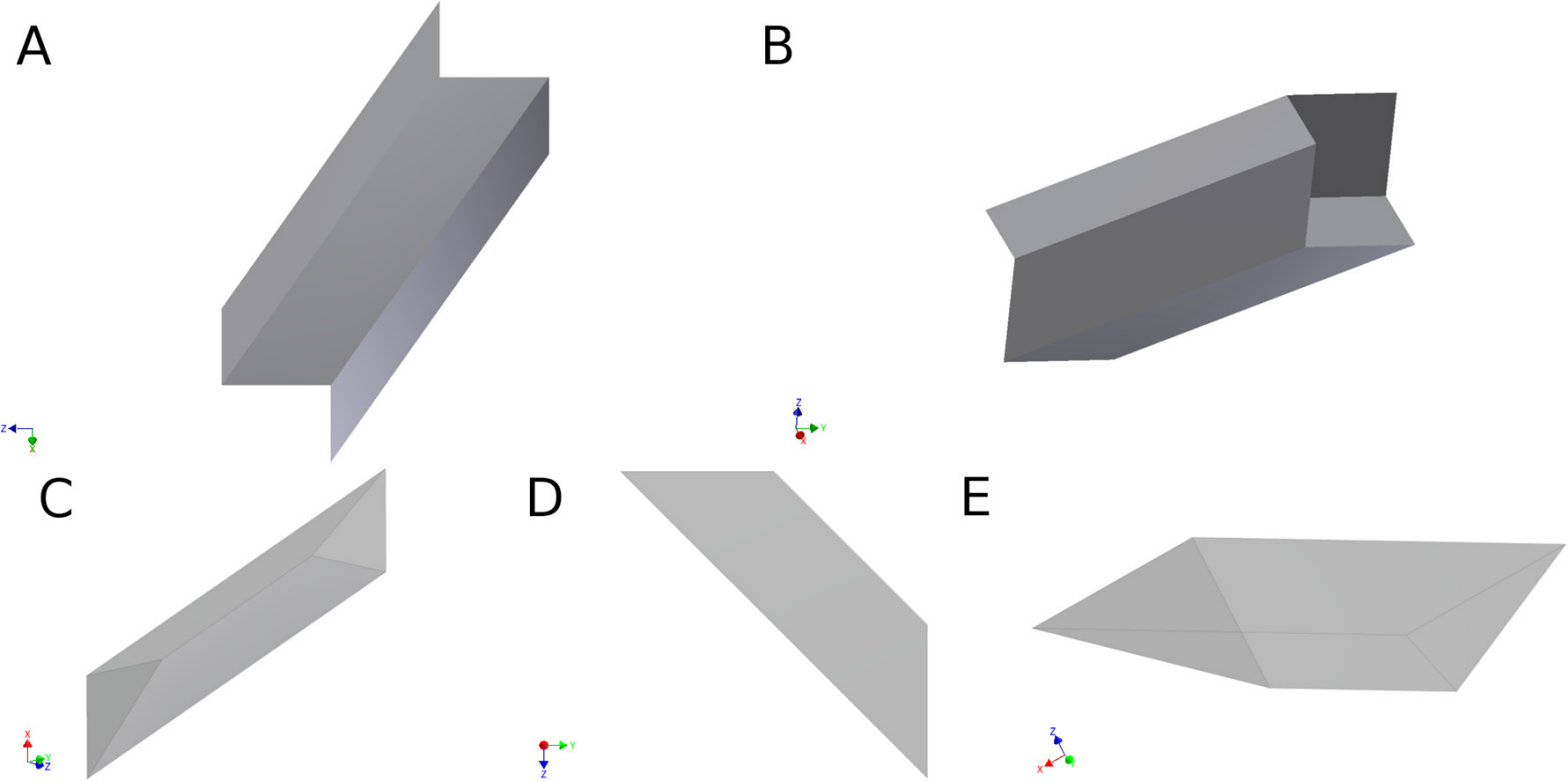
Slanted edge. (A) 3D edge sideways. (B) 3D edge at an angle to display the face where it connects to the node. Nodes are not shown to simplify the visualization. (C-D) Sketch of one of the portions that make up the fiber. (C) Shows that for every short fiber there are two of the long ones. (D) Shows a short side, which has a length of √3 and the longer one is 2√3. (D) Shows the triangular cross section of the fiber, yielding a higher number of longer fibers compared to the short ones.

The continuous edge weight is now used to calculate the connectivity strength for a fiber shown in Fig 5C. The surface area of the nodes is given by Eq A-6. The volume occupied by the fiber yields two long fibers for every short one (Fig 11), forming a triangular cross section instead of a square cross section as the slant on plane example. As the separation of the nodes, *M*, is increased one obtains the following number of fibers,

$$\begin{array}{c} M_{1}=1\\ M_{2}=2\\ M_{3}=3\\ \vdots \\ M_{M}=M \end{array}\begin{array}{c} \sqrt{3}+2(2\sqrt{3})\\ \sqrt{3}+2(3\sqrt{3})\\ \sqrt{3}+2(4\sqrt{3})\\ \vdots \\ \;\sqrt{3}+2((M+1)\sqrt{3}) \end{array}.$$ (A-14)

The mean length at any separation, *M*, of the voxels is given by

$$\bar{l}=\frac{\sqrt{3}Md+2\sqrt{3}(M+1)d}{3}=\sqrt{3}d\frac{3M+2}{3}.$$ (A-15)

**Fig 12:**
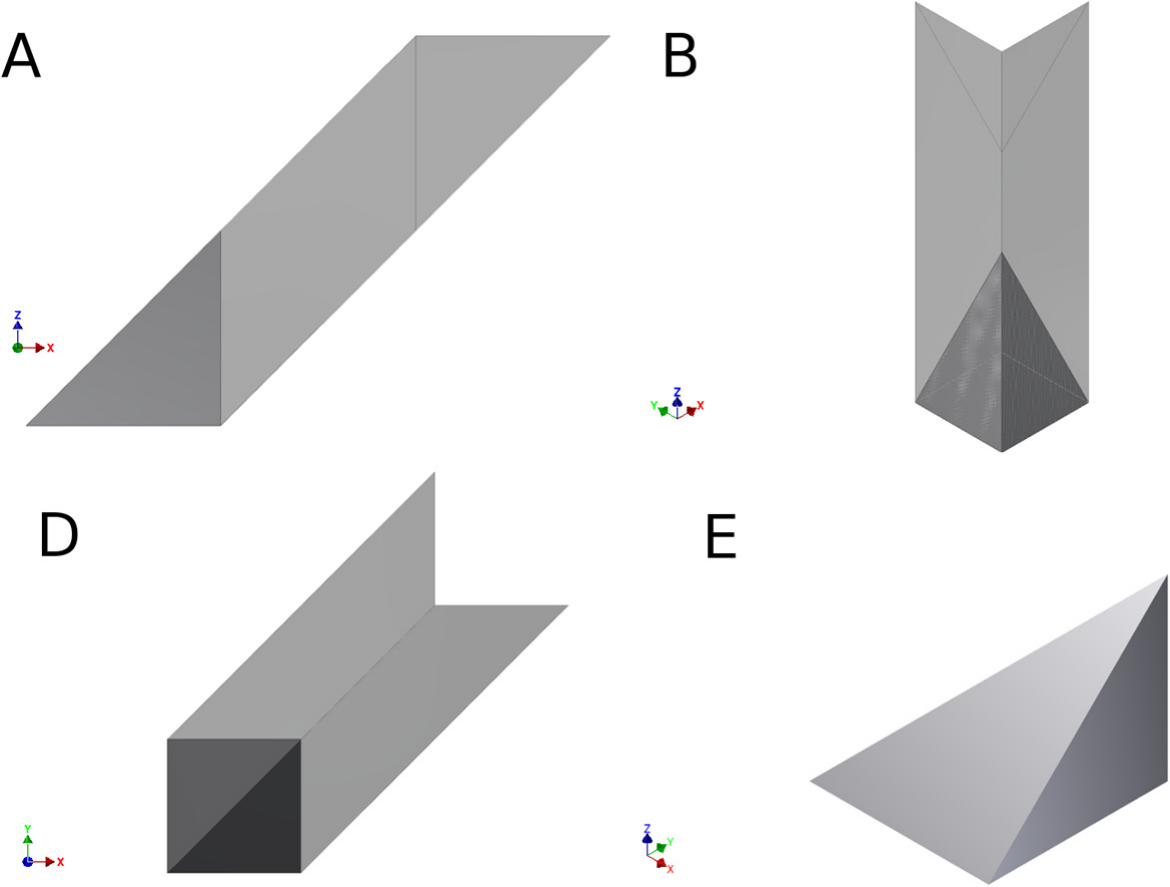
Sketch of the portion of the voxel (dark gray) adjacent to the node that contributes to the edge. (A) Shows the dark gray corresponding to the voxel above the node voxel on a sideways view. (B) Shows the voxel above the node voxel on a view along the z axis. (C) Shows to the voxel above the node voxel on a top view. (D) Sketch of the isolated piece of the voxel contributing to the edge. This volume is repeated along the fiber length except in middle nodes where the entirety of voxel contributes to the edge weight.

The occupied volume by the fiber on each voxel (Fig 12) is obtained by calculating the following

$$\int_{R}\;dV\approx R_{1},\;0<z<dy,\;0<x<d,\;0<y<dx.$$ (A-16)

The solution of Eq A-16 yields half of the contribution of each voxel to the edge and is obtained by,

$$\int_{R_{1}}dV=\int_{0}^{d}\left(\int_{0}^{xd}\left(\int_{0}^{dy}dz\right)dy\right)dx=d\;\int_{0}^{d}\;{\int_{0}^{dx}z|}_{0}^{yd}dy\;dx=d^{2}\int_{0}^{d}\frac{y^{2}}{2}|dx=d^{3}{\frac{x^{3}}{6}|}_{0}^{1}=\frac{d^{3}}{6}.$$ (A-17)

The total volume is twice of Eq A-17, yielding a result given by

$$\int_{R_{1}}dV=\frac{d^{3}}{3}.$$ (A-18)

At any separation, *M*, the number of voxels and the volume each voxel contributes to the edge weight is given by

$$\begin{array}{l} \begin{array}{c} separation\;\\ M_{1}=1\\ M_{2}=2\\ M_{3}=3\\ \vdots \end{array}\begin{array}{c} \left(\mathrm{number of}\;d^{3}/3\;\mathrm{voxels}\right),\;\mathrm{number of}\;d^{3}\;\mathrm{voxels}\\ (3,3,6),\;1\\ (3,3,6,6),\;2\\ (3,3,6,6,6),\;3\\ \vdots \end{array}\\ \begin{array}{cc} \;M_{M}=M & \;(3,3,6M),\;M \end{array}. \end{array}$$ (A-19)

Therefore, the total volume of region *R* becomes,

$$\int_{R}\;dV=\frac{d^{3}}{3}(6M+6)+Md^{3}=(3M+2)d^{3}.$$ (A-20)

Finally, the edge for the slant on a plane with a surface area of the nodes specified by Eq A-6, a mean edge length equal to Eq A-15 and the volume occupied by the fiber obtained by Eq A-20, yields an edge weight given by,

$$w(e)=\frac{1}{6d^{2}}\frac{1}{\sqrt{3}d\frac{\left(3M+2\right)}{3}}(3M+2)d^{3}=\frac{1}{2\sqrt{3}}\approx 0.289,$$ (A-21)

regardless of the physical length of the edge.
